# Common misinterpretations of statistical significance and *P*-values in dairy research

**DOI:** 10.3168/jdsc.2025-0835

**Published:** 2025-09-25

**Authors:** R. Laven, D.A. Yang

**Affiliations:** 1 Täwharau Ora–School of Veterinary Science, Massey University, Palmerston North, New Zealand 4442; 2 College of Veterinary Medicine, Nanjing Agricultural University, Nanjing, China 210095

## Abstract

Careful communication of results is integral to dairy research. However, many published studies contain misinterpretation of the results of statistical analysis, which can lead to conclusions being drawn which are not consistent with the data. Many of these interpretations have arisen because of a focus on *P-*values rather than on the potential range of effects that are compatible with the study data. This review focuses on 3 misinterpretations: the use of levels of statistical significance to compare results between or within studies, overinterpretation of nonsignificant results, and the use of “trend” to describe results that are “close” to a significance threshold. All of these misinterpretations can be avoided by paying more attention to the range of effects that are compatible with the data. Such a focus will have many benefits—not least, making it clearer when studies have insufficient power to accurately characterize their outcomes. Focusing on compatible effects is not a panacea but will improve statistical inference and provide more thoughtful descriptions of study outcomes.

## Highlights

• A difference in statistical significance is not necessarily biologically important.
• The absence of statistical significance does not mean there is “no effect.”
• Don't use trend/tendency to mean “near significant.”
• More data will not always support your initial findings.
• Focus on effect sizes and practical importance, not *P*-values.

## Graphical Abstract

**Figure:**
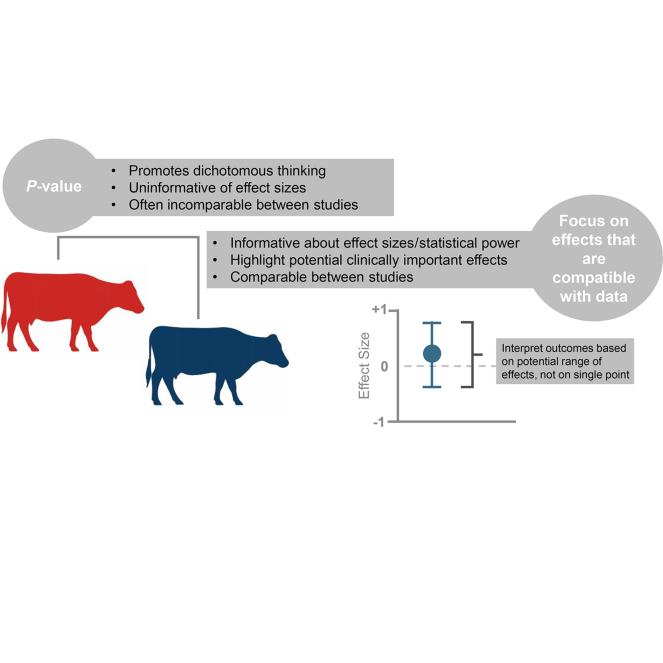
**Summary:** The focus in published dairy research on *P*-values has limited the value of such research by dividing results into either “significant” or “nonsignificant.” A focus on effect sizes will improve the interpretability and value of dairy research and reduce dichotomous thinking.

Statistics is an essential component of dairy research, playing an integral role in determining whether the conclusions from the data can “withstand close interrogation and independent validation” (Bello and Renter, 2018). To achieve this, we need meticulous study design, well-planned and conscientious data analysis, and careful communication of conclusions. These are all critically important and interlinked. When design or analysis are weak, misinterpretation becomes almost inevitable, and even high-quality studies can be undermined by careless or simplistic communication with researchers using stock phrases that lack nuance, or ignoring the outcome of the analysis to focus on whether an arbitrary probability threshold has been met.

This review focuses on the miscommunication of results in 3 areas. All are common in published dairy research and linked to a focus on *P-*values rather than effect sizes. These are (1) misinterpretation of a difference in levels of statistical significance between 2 groups; (2) misinterpretation of an outcome that is not statistically significant; and (3) misinterpretation of an outcome that has a *P-*value just above the “statistically significant” threshold. For all 3 areas, we set out the issues and provide examples of published papers with misinterpretation, before discussing how the results could have been better interpreted. It is important to note that we are not claiming that cited papers are examples of “poor-quality” research, just that they illustrate errors of interpretation. Indeed, our working assumption, unless otherwise stated, is that all research discussed is well-designed and analyzed, and therefore outcomes reported are unbiased reflections of the true effects.

## (1) Misinterpreting the Difference in Levels of Statistical Significance Between Two Groups

This involves drawing a sharp distinction between 2 sets of results (from the same or different studies) on the basis that one is statistically significant and the other is not. However, “the difference between ‘significant’ and ‘not significant’ is not itself statistically significant” (Gelman and Stern, 2006); that is, just because one set of results has a *P-*value >0.05, and the other does not, says nothing about whether a between-group difference exists. To identify whether a difference exists, one must formally statistically analyze that difference, not compare significance levels.

Our first example is a within-study comparison, Whay et al., 2005. These authors compared the effect of ketoprofen against placebo on nociceptive threshold (**NT**) in lame cows. Compared with baseline, cows given ketoprofen had significantly greater thresholds after treatment, whereas for cows given placebo the change was not significant. Whay et al. (2005) did compare between the two treatments at each time point using a *t*-test, but this “did not reveal any significant differences between the two groups.” Despite (or perhaps because of) this conclusion, their presentation of results focused on the within-group comparison. Their abstract stated that ketoprofen modulated lameness-associated hyperalgesia, whereas their discussion directly contrasted the significant ketoprofen effect with the nonsignificant placebo effect, downplaying the nonsignificant between-group difference by suggesting it was due to “small group sizes.” Thus, their focus on contrasting significance levels created a mismatch between their study objective and their analysis. Mason et al. (2022) analyzed the data reported by Whay et al. (2005) in relation to NT and concluded that although, on all 3 measurement days, mean NT was higher for ketoprofen-treated cows, the 95% CI for the difference between the NT of the 2 groups ranged from negative to positive; for example, on d 3, mean difference was 1.2 Newtons (95% CI −0.49 to 2.89). Thus, to use the term introduced in Gelman and Greenland, 2019, the results were “compatible” with the effect of ketoprofen on NT being anywhere from a small negative effect to a large positive one. Thus, the claim that Whay et al. (2005) demonstrated that treatment with ketoprofen increases NT is based on a flawed comparison of significance levels, which confuses a within-group comparison with a between-group one.

The paper by Whay et al. (2005) is a particularly good example of the persistence of misinterpretation (and thus the importance of avoiding it). Despite explicit acknowledgment in the paper that the between-treatment difference was “not statistically significant” and critiques of their interpretation by Laven et al. (2008) and Mason et al. (2022), Whay et al. (2005)'s finding continues to be misinterpreted and reported. For example, 20 years later, Wilson et al. (2025) cited Whay et al. (2005) as evidence that nonsteroidal anti-inflammatory drugs were useful for pain modulation in lame cows, despite also citing both critical papers.

An even more common form of within-study comparison by significance level is the focus on individual “significant” time points in a series. Such comparisons are an inevitable outcome of a modeling process in which, because either the effects of treatment or the treatment × time interaction are “significant,” there is then a focus on “significant” individual time points. The issue lies not in the detection of treatment × time interactions but in the presentation and interpretation of the results. For example, Sampson et al. (2009) measured serum Ca concentrations in bolus-treated and control cows after calving and reported that concentrations were “significantly higher (*P* <0.02; SE 0.06) for the bolus group versus the control group [only] at 13 hours.” As in many such studies, the value of identifying individual time points is unclear, as the focus of the abstract and discussion was the increase in overall Ca. However, their presentation of the results implies a meaningful divergence in the between-treatment differences at 12 and 13 h. Based on the figure presented by Sampson et al. (2009), we calculated that mean between-treatment difference at 13 h was 0.13 mmol/L (95% CI 0.02–0.24), whereas at 12 h it was 0.07 mmol/L (95% CI −0.06 to 0.2). The clear overlap between these estimated confidence intervals means that the difference at 13 h is compatible with our point estimate of the difference at 12 h. This conclusion comes with the caveats that our 95% CI are based on the assumptions that sample size is very large and that the figure represents model output. Unfortunately, Sampson et al. (2009) supplied insufficient data to calculate the degrees of freedom for the model, and the between-treatment difference at 13 h from the figure (0.13 mmol) is not compatible with the reported *P*-value of 0.02 given the SE of 0.06 (even assuming a large sample size). Presenting more data such as the sample size for each analysis and the SE and 95% CI for each effect estimate, and, where feasible, making full model outputs (i.e., regression coefficients, covariance matrices) available would solve this problem. However, simply presenting more data, so that readers can do the calculations that the authors should have done, does not solve the problem. Authors, irrespective of how much data they provide, should not emphasize differences in levels of significance without seeing whether those differences reflect the data.

The second scenario in which comparison between significance levels is incorrectly assumed to imply a significant difference is between-study comparisons. This is a common approach in the Introductions of published dairy research, with comparisons frequently being made without questioning whether the data from the 2 studies support the conclusion of a difference or even whether they are directly comparable. For example, Dai et al. (2023) claimed that data on the relationship between age and postpartum uterine involution in dairy cows were “inconsistent,” on the basis of one “statistically significant” study in Zebu cattle and one in Finnish dairy cows that was not. The main negative effect of such comparisons in an introduction is promoting the idea that outcomes can be summarized in this way. However, in a Discussion, comparing the current study's significance level with those of previous studies can result in conclusions being made about the current study that are not supported by the evidence.

For example, Zahra et al. (2006) investigated the effect of administering a monensin controlled-release capsule to late-pregnant cows, including effects on serum levels of nonesterified fatty acids (**NEFA**), and concluded that there “were no signiﬁcant treatment effects on serum NEFA.” They contrasted this to the “conflicting findings” of Duffield et al. (2003) that “administering monensin controlled-release capsules . . . signiﬁcantly reduced serum NEFA.” However, this comparison was based solely on significance levels, not on a formal statistical analysis. Both Duffield et al. (2003) and Zahra et al. (2006) reported estimated marginal means, SD or SE, and treatment group size, so we were able to calculate, using a *t*-test, the mean difference in treatment effects between the 2 studies at 1 wk before calving: 0.07 MEq/L (95% CI: −0.05 to 0.19)—meaning the data were compatible, with no difference, despite the “conflicting” claim. The caveat to this is that, as with Sampson et al. (2009) earlier, our analysis overlooks the model structures used in the original studies, but the parameters needed for a more robust comparison, such as regression coefficients and a covariance matrix for the coefficients, were not provided by either study, although, as demonstrated in the forest plot for a meta-analysis of the influence of monensin on NEFA (Duffield et al., 2008), our conclusion that the data were compatible, with no between-study difference, is robust.

Nevertheless, at least Duffield et al. (2003) and Zahra et al. (2006) provided some parameters that allowed direct comparison. The lack in many published papers of the data or parameters required for proper comparison is a key driver of between-study comparison by significance level. Another factor is differences in study design such as choice and categorization of predictors, modeling processes, and outcomes measured. An example of a between-study comparison where comparison beyond significance level is not possible because of different (but related) outcomes is that made by Carbonari et al. (2024). Those authors contrasted their finding that prostaglandin treatment resulted in “more efficient uterine involution” against that of Stephen et al. (2019), who reported no significant effect. However, it is impossible to analyze the claim by Carbonari et al. (2024) that the 2 results conflict, because they used uterine diameter at 28 d as their main outcome, whereas Stephen et al. (2019) analyzed the rate of uterine involution. This finding that 2 studies compared in a paper are not really comparable is not unusual in the dairy science literature. Our literature search for this review identified a large number of studies whose results were compared against previous studies using significance levels, but in almost all cases no direct comparison was possible, because of missing data or parameters, differences in study design, or differences in outcomes. This lack of standardized reporting between studies has been identified as a major factor restricting the application of meta-analyses in dairy science (Lean et al., 2009, 2016). Sophisticated, targeted techniques can allow meta-analyses to overcome such issues, but comparing significance levels is not part of robust meta-analysis. (Indeed, *P-*values from the underlying studies play no role in meta-analysis.) When comparing results between studies, authors should never simply report conclusions based on differences in significance level. If studies are directly comparable, such as Zahra et al. (2006) and Duffield et al. (2003), then authors should use the data presented to calculate the range of compatible differences (with the appropriate caveats, especially where the original analyses used a complex statistical approach). Where differences between studies prevent a direct comparison, authors wishing to compare results should present data from the studies being compared in point estimate (CI) form and compare the conclusions from those estimates.

## (2) Interpretation of an Outcome That Is Not Statistically Significant

Recognizing that comparison of significance levels is flawed emphasizes the need for accurate interpretation of “nonsignificance.” A statistically nonsignificant result does not mean that no effect exists. Rather, it means that, assuming the null hypothesis is correct, the probability of getting a difference at least as extreme as the result obtained by the researchers is greater than the significance threshold (i.e., for most studies, *P* > 0.05). Or, using Greenland's terminology, a nonsignificant result means that the data are “compatible” with no effect. However, it is important to note that such data are compatible with a range of results, not just “no effect.” The range of compatible results can be determined by calculating a confidence interval. As Greenland states, “all values in a conventional 95% [confidence] interval can be described as highly compatible with the data under the background statistical assumptions, in the very narrow sense of having *P* > 0.05 under those assumptions” (Gelman and Greenland, 2019).

Thus, when reporting nonsignificant effects, it is important not to focus on whether the data are compatible with no effect, but on the range of effects with which the data are compatible. Doing this avoids claims of “no effect” when there is simply insufficient data, as when confidence intervals are wide and include both biologically (clinically) important positive and negative effects. In addition, when confidence intervals are narrow and not compatible with biologically or clinically important effects, focusing on the range of compatible effects can provide support for the conclusion that it is unlikely that there are any meaningful effects.

Leal et al. (2025) investigated the effects of preweaning milk allowance on multiple outcomes, including first-service conception rates (**FSCR**). They concluded that preweaning milk allowance had “no effect” on FSCR. However, this overstates the certainty around this conclusion and ignores the range of effects compatible with their data. Unadjusted FSCR were 73% and 63% for calves fed 8 L and 4 L, respectively, a difference of 10% that would be important if it reflected the underlying true difference. Leal et al. (2025) used a multivariable logistic regression but reported neither adjusted FSCR from the model nor the confidence interval for the difference between those 2 rates. Nonetheless, we can back-calculate both of these, as the authors provided the SEM and the *P*-value for the difference from the model. Our calculation identified that the mean difference in FSCR from the model was 7.7% (95% CI −6.8 to 22.2); that is, the outcomes compatible with the data ranged from a large decrease in FSCR to a very large increase. Thus, rather than demonstrating “no effect,” the analysis actually demonstrated that the authors could not make any robust conclusions about the likely influence of preweaning milk allowance on FSCR. Thus, the speculation in their discussion as to why they did not, contrary to their hypothesis, find an effect of milk allowance on reproductive performance is not justified, as they did not find “no effect”; no difference was just one point in a very wide range of points compatible with their data.

Leal et al. (2025) do state that the number of calves in each group was “limited to detect differences in conception rate.” Such statements regarding low study power are common in studies where large differences in outcomes turn out to not be statistically significant. There are many issues with such statements (Button et al., 2013; Heinsberg and Weeks, 2022), but for this review the key problem is that lack of power limits a study's ability to support the conclusion that there is no meaningful effect. For example, if Leal et al. (2025) had found no difference in FSCR and still (for simplicity) had the same SEM, we would have a mean difference of 0% with a 95% CI of −14.5 to 14.5. In that case, our conclusion would be that, despite finding no between-group difference in FSCR, evidence was insufficient to determine whether there was truly an effect (either positive or negative) or none. Claiming that we had shown no effect would still be just as wrong as with a mean difference of 10—although, unfortunately, it might be more persuasive for the reader, because of the natural reader focus on point estimates.

It is sometimes suggested that nonsignificant effects should be described as “no evidence for treatment differences” (Bello and Renter, 2018), but this is incorrect. This can be seen using an imaginary example in which an appropriately designed and analyzed study is repeated multiple times and yields the same mean and SEM every time. For a study in which the outcome has a point estimate of 0, putting results together narrows the confidence interval around the estimate of no difference; that is, support for the hypothesis that the true difference is 0 increases. In contrast, starting with a point estimate of 10, putting results together narrows the confidence interval around the estimate of a difference of 10. Thus, as we add the results from more repetitions, we get to the point where the data are no longer compatible with “no effect”: in other words, we now have “evidence” of treatment differences. Thus, unless the point estimate is 0 (which is very rarely true), nonsignificant outcomes do provide evidence in favor of treatment differences. This can be seen in the real-world situation in meta-analyses, when combining nonsignificant studies together can identify statistically significant effects (Lean et al., 2009). We recommend avoiding blanket terms such as “no evidence of treatment differences” and “no significant treatment differences,” as, although better than “no difference” or “no effect,” they still focus on whether *P* is greater or less than 0.05. Instead, authors should focus on the range of outcomes that are compatible with their data. In some cases, such as Leal et al. (2025), this means simply stating the mean difference and CI and concluding that data are compatible with such a wide range of plausible effects that making any claim is unjustified. In other cases, our conclusions can be more nuanced. For example, Van Schyndel et al. (2021) reported that pegbovigrastim had no “significant effects on the incidence of subclinical mastitis.” The odds ratio (**OR**) was 1.0 (95% CI 0.82–1.2); for a control incidence risk of 13%, this is equivalent to an incidence risk of 11% to 15% for pegbovigrastim-treated cattle. Thus, this conclusion could have been better stated as “The data are not compatible with a clinically meaningful change in subclinical mastitis risk.” Conclusions around the meaning of “no effect” depend explicitly on the understanding of what is biologically important, and are not dictated by *P-*values or simple formulas that can be applied to every situation. This approach is similar to that used to determine limits-of-agreement and in equivalence testing (Altman and Bland, 1983; Lakens, 2017). Thus, by using confidence intervals alongside specialist knowledge, we can maximize the value of a finding of “no effect.”

## (3) Interpretation of an Outcome That Has a *P*-Value Just Above the “Statistical Significance Threshold”

The lack of nuance when reporting a finding of “no effect” has led many dairy scientists to try to avoid having to report “no effect” by introducing a second threshold above their first. This is commonly written as, “The threshold for significance was set at *P* ≤ 0.05, and trends were reported at *P* < 0.10,” or similar. Thus, in the abstract and discussion, the authors refer to trends or tendencies when there is a failure to meet the designated “significance” threshold. This is an error which is “neither trivial nor merely semantic” (Gibbs and Gibbs, 2015). First, a “trend” or a “tendency” implies multiple results pointing in one direction, which can never be true of a single *P-*value. Furthermore, implying that a value close to, but above, the significance threshold points toward significance should also imply that values close to, but below, the significance threshold (e.g., 0.04) point toward nonsignificance. Yet, unsurprisingly, we have never seen this done. This is probably because the term “trend” is being used to imply that if the study had more power (more animals), the result would have been statistically significant. This assumption is consistent with the imaginary scenario we discussed earlier, where increased numbers narrowed confidence intervals and increased precision around the estimate of the true. However, this scenario required repetition of the same result (same point estimate, same SEM), a scenario that is extremely unlikely. This is because, in subsequent studies, random (unpredictable) variation in *P*-values and their associated confidence intervals will occur (Wood et al., 2014), because independent studies are effectively a random sample of the true state of nature (Berk and Freedman, 2003). However, we can calculate the probability that increasing numbers lower *P*-values, as this probability is dependent on the *P*-value of the original study and the relative amount of additional data (Wood et al., 2014). For a *P*-value of 0.08, if we added 20% more data and then reanalyzed all the data together (both new and old), our subsequent second *P*-value would be >0.08 ∼35% of the time and >0.05 54% of the time. If we added 100% more data, the second *P*-value would be >0.08 ∼23% of the time and >0.05 30% of the time. Simulations using expected effect size and sample size can further clarify uncertainty by showing how, given a properly selected model, increased sample size narrows 95% CI, thereby excluding biologically unimportant effects or confirming that such effects are the only effects compatible with the data. It is important to remember that these values are long-term averages; at the individual study level, we do not know what the effect of adding more data will be.

One simple solution to avoid the use of “trend” is to increase the *P*-value threshold (e.g., from 0.05 to 0.1). This may be the best solution for preliminary exploratory research, but it is important to remember that a *P*-value of 0.1 is effectively as surprising as tossing a fair coin 3 times and getting 3 heads (i.e., it provides only 3.3 bits of information against the null hypothesis; Greenland, 2019). Furthermore, simply swapping thresholds does not avoid “dichotomization by *P*-value” (Greenland, 2019). The better solution for avoiding use of “trend” is to focus on the range of effects compatible with the data. If this range includes biologically or clinically important effects, these can be reported alongside the whole range of compatible outcomes.

In their paper describing their study of risk factors associated with *Mycoplasma bovis*, Haapala et al. (2021) use the term “trend” on multiple occasions to describe results where *P-*values were close to but greater than 0.05. For example, they state that a “slight trend can be seen” for increased risk of a farm being infected after insemination with a known *M. bovis*–positive bull. The OR was 3.7 (95% CI 0.79–17.3); thus the data were compatible with a small and non-biologically important decrease in odds and a very large increase (i.e., if the rate of insemination with a known *M. bovis*–positive bull on uninfected farms was 16.5%, the rate of use of such bulls on infected farms would be between 14% and 78%). Thus, although Haapala et al. (2021) demonstrated that a meaningful decrease in infection risk from using a *M. bovis*–positive bull was not compatible with their data, their study lacked the power to accurately determine the likely influence of such insemination, even if, by chance, they had demonstrated “statistical significance.” Furthermore, as their model's *P-*value was 0.096, even doubling the data would be expected to result in a *P-*value >0.05 36% of the time (Wood et al., 2014). Similar concerns apply to almost all of the risk factors for *M. bovis* infection identified in their final model, with all but herd average milk yield having OR with wide 95% CI. This lack of precision is obscured by the use of “trend.” Their results also identify the futility of changing thresholds as an alternative to using “trend” or “tendency.” In their final model the effect of cattle purchase on the odds of infection had a *P-*value of 0.103 (“nonsignificant” using a threshold of 0.1); however the model OR was 2.98 (95% CI 0.8–11.06), so their data are compatible with cattle purchase having an equally great (or greater) influence on the odds of infection as any of their risk factors with *P* < 0.1.

## Overview

This review has focused on the interpretation of statistical outputs rather than study design and analysis. It is important, at this point, to emphasize again, as we did at the start, that these are absolutely critical; indeed, Greenland specifically argues for the term “compatibility” interval (Gelman and Greenland, 2019) because the “‘compatibility’ label offers no false confidence and no implication of complete uncertainty accounting.” Nevertheless, we believe that our focus on interpretation is important, as the rote usage of *P-*values as *the* determinant of outcome without actually looking at the effects identified by the analysis has led to significant and persistent misinterpretation and misunderstanding.

The focus on *P-*values or (more accurately) *P-*value thresholds leads to all 3 of the concerns discussed. In all 3 cases, using confidence intervals to identify the range of effects alerts the authors (and their readers) to the fact that the outcome of a statistical analysis is not just a single *P-*value but a whole range of potentially useful parameters (Amrhein and Greenland, 2022). Focusing on the range of compatible effects rather than significance level should stop authors claiming that there are differences between studies just because they have differences in significance level, as well as preventing claims of “no effect” when the study simply lacks the power to precisely determine that effect. Furthermore, “compatible intervals” can be used to replace the dichotomous thinking driving the use of “trend” and to demonstrate that the claim of “no effect” is actually meaningful because the interval includes only biologically unimportant effects. All of these changes result in clear improvement in the communication of study outcomes.

However, using confidence (compatibility) intervals is not a panacea. Their usefulness depends on good study design and robust analysis, and they have many of the same issues as *P-*values. These include being calculated in the same way as *P-*values and thus being subject to similar misinterpretations (Greenland et al., 2016). In particular, it is often claimed that for every 95% CI there is a 95% chance that the true value lies somewhere within that interval (Gelman and Greenland, 2019). However, the 95% claim is about outcomes from a large number of studies. It refers to how often, on average, a computed 95% CI would contain the true value “if all the assumptions used to compute the intervals were correct” (Greenland et al., 2016). For an individual confidence interval, all we know is that either it includes the true value, or it does not. Nevertheless, despite these issues, the use of confidence intervals improves statistical inference and reader understanding of study outcomes (Amrhein and Greenland, 2022).

Much of our argument also applies to “statistically significant” effects. Simply reporting *P-*values is as limited for these effects as it is for nonsignificant outcomes. We need to know effect size and practical importance, rather than the probability that we could have obtained test statistics as large as or greater than our point estimates, assuming there was no difference.

However, although evaluating ranges rather than point estimates reduces dichotomous thinking, it does not eliminate it (Greenland et al., 2016). We need to ensure that we do not continue to separate results on the basis of whether our confidence interval is compatible with no effect or not. As we discussed earlier, we think this is particularly important where repeated measures are made over time, and small differences can determine whether a confidence interval includes 0 or not. For this sort of time series, we need a better way of reporting results without, as Sampson et al. (2009) and many similar papers do, implying that results which are compatible with no effect are necessarily different from those which are not. We recommend reporting the treatment effect in terms of both the rate of change (slopes) and the cumulative effect over time (main effect at specific times). If it is necessary to contrast main effects across time points, formal statistical tests must be carried out.

Increased use of confidence intervals should reduce the number of studies that do not report them or the parameters necessary to calculate them. On many occasions we were unable to compare between studies or to properly evaluate claims of “no effect” because such data were absent. We recommend that editors should require papers to include such information, especially for complex models for which outcomes from the model, such as marginal means, are different from raw data.

Not all analyses routinely produce confidence intervals. However, methods are available to calculate such intervals for many analyses, including nonparametric tests (Elkins et al., 2022). If no method of producing a confidence interval is available or interpretation is difficult, then identifying an alternative test that can produce an interpretable outcome is often possible (for example, substituting an ordinal regression for a Wilcoxon Mann-Whitney test; Harrell, 2001). However, it is important not to overstate the importance of this problem; for most papers we examined for this analysis, the principal problem was not reporting confidence intervals but the use of statistical tests that did not produce them.

### Calls to Action

A rote focus on *P-*value thresholds has resulted in many authors of dairy science papers misinterpreting their study outcomes. Focusing on point estimates and *P-*values ignores practical importance. Reporting the range of compatible effects rather than *P-*values provides a more nuanced and useful focus, especially on the key question of “whether the effect estimates represent effect sizes substantial enough to be of practical importance” (Amrhein and Greenland, 2022). We recommend that editors of dairy science journals consider adopting the following minimal reporting standards for all statistical analyses: (1) report the sample size for each analysis; (2) provide the SE and 95% CI for all effect estimates (e.g., mean differences, OR) from the models, specifying the method of CI computation if not derived from SE using normal approximation; (3) make full model outputs (i.e., regression coefficients, covariance matrices) or, where feasible, processed data ready for modeling, available in a public repository to enable readers to compute contrasts or perform further analyses; (4) discuss the practical importance of effect estimates alongside CI, rather than focusing on *P-*values. We also recommend that editors ask reviewers to refrain from asking for *P-*values where they are not reported but estimates and 95% CI are.
